# Cancer Nanomedicine: Emerging Strategies and Therapeutic Potentials

**DOI:** 10.3390/molecules28135145

**Published:** 2023-06-30

**Authors:** Manman Xu, Xinpu Han, Hongtai Xiong, Yijie Gao, Bowen Xu, Guanghui Zhu, Jie Li

**Affiliations:** 1 Department of Oncology, Guang’anmen Hospital, China Academy of Chinese Medical Sciences, Beijing 100053, China; xummjournal@163.com (M.X.); a18329703725@163.com (X.H.); xht1799243721@163.com (H.X.); xubw2018@126.com (B.X.); zhugh0822@163.com (G.Z.); 2 Department of Integrative Medicine Cardiology, China-Japan Friendship Hospital, Beijing 100029, China; gaoyijiee@163.com

**Keywords:** cancer, nanomedicine, therapeutics, drug delivery, targeting

## Abstract

Cancer continues to pose a severe threat to global health, making pursuing effective treatments more critical than ever. Traditional therapies, although pivotal in managing cancer, encounter considerable challenges, including drug resistance, poor drug solubility, and difficulties targeting tumors, specifically limiting their overall efficacy. Nanomedicine’s application in cancer therapy signals a new epoch, distinguished by the improvement of the specificity, efficacy, and tolerability of cancer treatments. This review explores the mechanisms and advantages of nanoparticle-mediated drug delivery, highlighting passive and active targeting strategies. Furthermore, it explores the transformative potential of nanomedicine in tumor therapeutics, delving into its applications across various treatment modalities, including surgery, chemotherapy, immunotherapy, radiotherapy, photodynamic and photothermal therapy, gene therapy, as well as tumor diagnosis and imaging. Meanwhile, the outlook of nanomedicine in tumor therapeutics is discussed, emphasizing the need for addressing toxicity concerns, improving drug delivery strategies, enhancing carrier stability and controlled release, simplifying nano-design, and exploring novel manufacturing technologies. Overall, integrating nanomedicine in cancer treatment holds immense potential for revolutionizing cancer therapeutics and improving patient outcomes.

## 1. Introduction

Cancer continues to be a leading cause of mortality on a global scale, necessitating the pursuit of innovative and highly effective therapeutic strategies [1]. Over the years, cancer therapeutics has evolved significantly, comprising various approaches, including surgery, radiotherapy, chemotherapy, and immunotherapy. While surgical intervention and radiotherapy primarily address ear-stage, localized, non-metastatic cancers, chemotherapy and immunotherapy are usually employed in treating tumors resistant to surgery or irradiation or those presenting with metastases [2]. Despite their recognized effectiveness at curbing the proliferation of cancer cells, these drugs often adversely affect rapidly proliferating normal cells, such as those located in hair follicles, bone marrow, and the gastrointestinal tract, leading to severe short-term and long-term side effects [3,4]. Moreover, certain drugs are impeded by the blood–brain barrier, preventing them from effectively reaching tumor tissue [5]. Low drug delivery efficacy to tumors inevitably leads to cellular resistance [6] and rapid penetration into healthy brain tissue. Therefore, developing novel, targeted drug delivery strategies is paramount to striking a balance between anti-tumor drug efficacy and side effects [7].

In recent years, nanomedicine has garnered substantial attention due to its potential to address some of the limitations inherent to traditional therapeutic strategies, offering prospects for enhanced efficacy and minimized side effects. Nanomedicine, typically with sizes ranging from 1 to 100 nanometers, possesses distinct physicochemical properties that can be fine-tuned to optimize their interactions with biological systems [8]. These properties can be leveraged to improve tumor targeting, bolster immune regulation, and enhance drug delivery, tackling some primary challenges in tumor immunotherapy. Liposomes, polymeric drug conjugates, and polymeric micelles are the most extensively investigated nanomedicines of clinical relevance. These tailor-made nanomaterials facilitate the delivery of chemotherapeutic drugs to tumor sites, thus minimizing harm to non-target tissues, reducing multidrug resistance, assisting in tumor diagnosis and imaging, and improving therapeutic indices [7,9].

In contrast to traditional small-molecule drugs, nanomedicine can carry both hydrophilic and hydrophobic drugs, preventing rapid blood clearance, supporting sustained drug release, extending plasma circulation half-life, enhancing tumor accumulation, improving bioavailability, and mitigating toxic effects, all of which contribute to amplifying anti-tumor capacity [10]. However, potential toxicity concerns associated with certain nanomedicines necessitate a comprehensive regulatory evaluation before their implementation as anti-tumor therapeutic agents [11]. For instance, carbon nanotubes or dendrimers have yet to receive Food and Drug Administration (FDA) approval or initiate clinical trials due to unresolved toxicity issues [12]. Consequently, nanomedicine must surmount various hurdles to achieve efficient tumor-targeted drug delivery. This review aims to delve into the mechanisms and benefits of nanoparticle (NP)-mediated drug delivery, the role of NPs in tumor diagnosis, imaging, and therapy, the current challenges faced, and the potential future directions.

## 2. Nanoparticle-Mediated Drug Delivery

### 2.1. Mechanisms of Nanoparticle-Mediated Drug Delivery

#### 2.1.1. Passive Targeting

**Figure 1:**
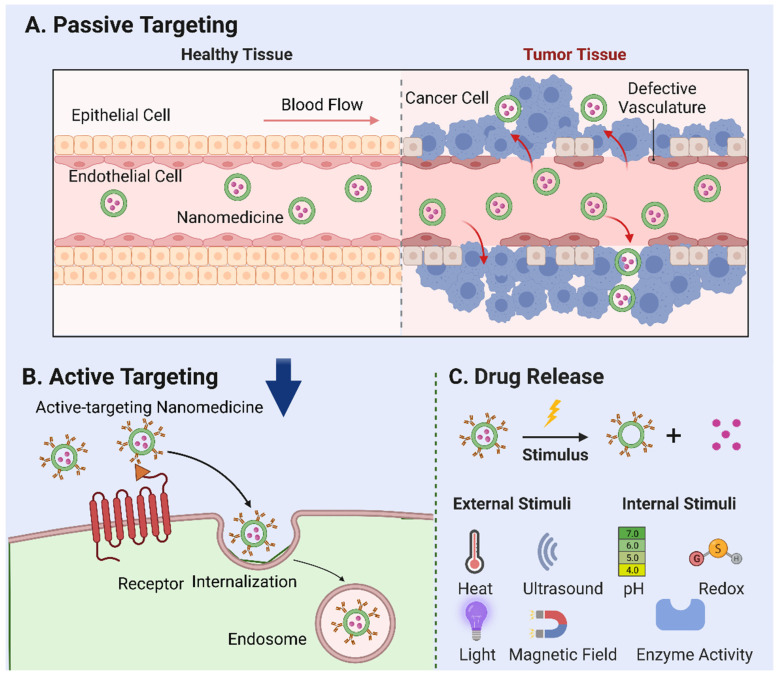
Mechanisms of nanoparticle-mediated drug delivery. (**A**) Passive targeting: the accumulation of nanomedicine in tumor tissues based on their specific physical and chemical properties—a phenomenon known as the “enhanced permeation and retention (EPR) effect”. (**B**) Active targeting: the use of ligands or antibodies to target tumor cell surface receptors. Following internalization, endogenous particles are transported to the nuclear endosome, where further particle processing and drug release occur. (**C**) External stimuli, such as temperature, ultrasound, light, magnetic fields, and internal factors, including pH, redox reactions, and enzyme activation, that can be employed to trigger the release of drugs loaded in nanoparticles.

Tumor vasculature, marked by a high ratio of proliferating endothelial cells and swift angiogenesis, exhibits unique pathophysiological traits. The rapid expansion of tumor tissues prompts irregularities in cell architecture, such as an increased cell curvature, insufficient neovascular outer membrane cells, and basement membrane deformation. Together with poor lymphatic reflux in tumor tissues, these irregularities foster the accumulation of NPs that penetrate through tumor capillary “gaps” and infiltrate tumor tissues—a phenomenon known as the “enhanced permeation and retention (EPR) effect” [13,14,15]. The EPR effect forms the foundation for passive targeting [16,17], a key mechanism for the tumor accumulation of prospective nanocarriers (Figure 1A).

The effects of nanomedicine within the body are considerably influenced by particle size. For instance, NPs of less than 5 nm are susceptible to quick renal filtration due to their hydrophilic nature. In comparison, NPs exceeding 200 nm often accumulate rapidly in healthy organs or are swiftly absorbed by macrophages [18,19]. Nanocarriers with particle sizes ranging between 5 and 250 nm can infiltrate leaking tumor vessels and accumulate effectively over time, given the absence of functional lymphatic drainage in tumor tissues [16,17]. Surface properties, such as charge and hydrophobicity, are vital in influencing protein photochemical changes and cellular uptake rates. Unlike hydrophobic and positively charged particles, neutral or negatively charged and hydrophilic particles demonstrate lower non-specific internalization rates and shorter in vivo circulating half-lives [20,21]. Stability, another key determinant of NPs’ circulation duration, is equally critical for efficient tumor targeting. Studies have shown that drug leakage from stable NPs often occurs before they reach their target [22,23,24], rendering premature drug release a significant factor impacting tumor targeting efficiency. The efficacy of passive targeting relies on tumor biology, including factors such as the extent of vascular and lymphatic vessel generation, perivascular tumor invasion, and intra-tumor pressure. These elements, along with the physicochemical properties of NPs, determine the efficiency of passive nanomedicine targeting. In addition, the efficiency of targeting heavily relies on blood circulation time [25]. Indeed, for systemically administered nanomaterials intended as drug delivery systems, kinetically slow transportation into the diseased tissues due to vascular barriers necessitates a relatively longer circulation time to increase the chance of crossing the vascular wall. One of the critical hindrances to prolonged blood circulation is the rapid clearance of nanomedicine by the reticuloendothelial system (RES).

To counteract this issue, steric stabilization techniques, such as PEGylation, have been introduced. This method exploits the principle of steric repulsion to retard RES clearance and prolong circulation. For example, the nanotechnology field leverages PEG to create ‘stealth’ drug carriers granting prolonged circulation times and decreased recognition and clearance by the mononuclear phagocyte system (MPS) [26]. Today, most nanomedicines approved for clinical use contain PEG, and it has been instrumental in recent advancements, such as the two mRNA-based COVID-19 vaccines, which are delivered by PEGylated lipid nanoparticles [26]. However, despite its pervasive use, it has become clear that PEG is not as immunologically inert as initially thought. Some studies increasingly indicate that the repeated systemic injection of PEGylated drugs can trigger immunogenic responses [27,28,29], which may result in hypersensitivity reactions and compromised therapeutic efficacy. Furthermore, anti-PEG antibodies have been detected in healthy individuals who have not previously received PEGylated drugs, hinting at a complex immunological landscape yet to be fully understood. Recent studies have unveiled the intergenerational inheritance of anti-PEG antibodies, shedding light on the potential long-term ramifications of PEGylated nanomedicines [29]. In addition, high titers of anti-PEG antibodies have been associated with adverse events, such as non-infectious fever after the administration of PEGylated therapeutics, and shown to interact with PEGylated liposomes [30]. Given the above circumstances, the clinical use of PEG-conjugated therapeutics has been impeded by its immunogenicity. A novel approach mitigates this issue by incorporating gangliosides into PEGylated liposomes, significantly reducing the production of anti-PEG IgM and subsequent rapid clearance of the drug [31]. This strategy promises to sustain the therapeutic efficacy of PEGylated liposomes, particularly upon repeated administration. Thus, researchers are currently exploring novel alternatives to address these emerging challenges. Such alternatives offer a promising pathway toward minimizing the immunogenicity of nanoparticle-mediated drug delivery, thereby enhancing their therapeutic potential.

#### 2.1.2. Active Targeting

An important subtype of active targeting, cell targeting, is an emerging field in nanomedicine. Targeting specific cells such as cancer cells, immune cells, or even stem cells can potentially improve treatment outcomes in various diseases [32]. This approach involves the design of NPs with specific ligands that can recognize and bind to receptors unique to the target cells (Figure 1B) [33]. A vital component of this strategy is the ligand-mediated recognition of the target substrate receptor [34]. Interactions between the ligand and target can induce intramembrane folding, leading to NP internalization via receptor-mediated endocytosis. Following internalization, endogenous particles are transported to the nuclear endosome, where further particle processing and drug release occur. This cell-specific delivery can improve therapeutic efficiency and reduce side effects by ensuring that the drugs primarily affect the cells of interest. Numerous studies underline the potential of cell targeting, showcasing a significant enhancement in cellular NP uptake in vitro. For example, anti-HER2 monoclonal antibody-modified, actively targeted magnetic NPs produced 10–30-foldhigher tumor tissue concentrations than did their non-targeted counterparts [35]. Similar results were observed with folic acid-modified layered double hydroxides and paclitaxel-loaded nanocarriers [36,37]. By selectively targeting cancer cells and cells adjacent to tumor blood vessels (such as angiogenic endothelial cells), cell targeting can disrupt the blood supply to cancer cells, instigating hypoxia and necrosis. Moreover, this strategy can augment the efficacy of nanocarriers bearing conventional chemotherapeutic drugs. However, the NPs designed for cell targeting must satisfy the prerequisites for passive targeting, including having a suitable particle size, adequate stability, prolonged blood circulation, and efficient drug retention [10].

However, cell targeting is not without its challenges. The complexity of biological systems poses significant hurdles. Upon administration into biological fluids, nanocarriers attract proteins onto their surfaces, forming an adsorption layer called the protein corona [38]. This protein layer significantly influences the biological behavior of nanocarriers by altering their physicochemical properties and can ultimately control their in vivo fate. This process significantly impacts nanocarriers’ stability, targeting capacity, pharmacokinetics, and toxicity. Therefore, the physiological function of nanoparticles heavily depends on the characterization of the proteins forming the corona [38]. Moreover, protein corona is a Janus-like entity that can positively and negatively impact NP-based therapeutics [39]. On the one hand, protein corona can influence blood circulation, accumulation and penetration at targeting sites, cellular uptake in tumor-targeting delivery, and interactions between NPs and immune cells for immunotherapy. On the other hand, protein corona can also cause unpredictable in vivo behaviors due to the absorption of biomolecules on NPs. For instance, protein corona can reduce the interactions between NPs and the cell membrane by covering the initially modified ligands or presenting an unfavorable steric effect, which decreases cellular uptake [39]. In summary, an in-depth understanding of protein corona and its complex interactions with NPs is essential in designing effective nanocarrier systems for therapeutic delivery.

After discussing cellular targeting, another significant component of active targeting, known as transcytosis nanomedicine, warrants our attention [40]. In contrast to the classical active targeting mechanism, which predominantly involves the binding of nanomedicine to cell surface receptors, transcytosis nanomedicine takes a step further. It is designed to be transported across cells, exploiting cellular machinery to transport nanomedicine from one side of the cell to the other. This process is beneficial in crossing biological barriers that are otherwise hard to penetrate, such as the blood–brain barrier or that of specific dense tumoral tissues. The application of transcytosis nanomedicine becomes especially significant in cancer therapy, where the tumor microenvironment (TME) often presents a formidable challenge to conventional drug delivery methods. By utilizing transcytosis nanomedicine, we can enhance the accumulation of therapeutic agents in tumor tissues, improving the efficacy of the treatment while minimizing systemic toxicity. Several strategies can be employed to design transcytosable nanomedicines, including the use of targeting ligands that bind to specific cell-surface receptors triggering endocytosis [41] and the design of nanocarriers that can escape endosomal entrapment to reach the cytosol, promoting exocytosis on the other side of the cell [42]. Furthermore, they can also be engineered to be responsive to various stimuli present in the TME, thereby enhancing their transcytosis efficiency [40]. To fully leverage the potential of transcytosis nanomedicine, ongoing research efforts are dedicated to understanding the molecular mechanisms underlying transcytosis and devising more efficient and specific nanocarrier systems. However, the complexity of transcytosis, combined with the heterogeneity of TME, necessitates a careful and nuanced approach to designing and applying these nanomedicines.

#### 2.1.3. Drug Release

Following the internalization of the nanomedicine, they are transported to the nuclear endosome for further processing and drug release. This process involves the disassembly or degradation of the nanocarrier and the subsequent release of the encapsulated therapeutic agent. The release mechanism can be either passive, which depends on the diffusion or dissolution of the drug, or active, which is triggered by specific stimuli within the cellular or TME.

In addition to the findings already outlined in our paper, several relevant studies in the stimuli-responsive nanomedicine field should be considered. Stimuli-responsive nanomedicines represent an innovative approach towards enhancing drug delivery efficiency, characterized by the capacity to respond to specific physical or biological triggers and subsequently control drug release profiles spatially, temporally, and in terms of dosage [43]. External stimuli such as temperature, ultrasound, light, and magnetic fields, as well as internal factors, including pH, redox reactions, and enzyme activation can be employed to trigger the release of drugs loaded in NPs [44] (Figure 1C). As elucidated by Mura et al. [43], these systems have the potential to regulate drug biodistribution and improve targeted delivery to diseased areas, thereby addressing the challenge of non-specific cell and tissue biodistribution that often alters drug efficacy. These nanoreactors remain inactive in normal tissues but are activated by the acidic TME, triggering the release of reactive species to induce oxidative stress and suppress the antioxidant abilities of cancer cells, thus leading to synergistic tumor ablation [45]. A study [46] worked on thermoresponsive liposomes for enhanced anti-cancer efficacy. They observed the more significant cytotoxicity of cisplatin-loaded thermoresponsive liposomes at higher temperatures, aligning with the elevated temperature at tumor sites. In recent research [47], a method was developed using temozolomide-loaded magnetic nanoparticles combined with an alternating magnetic field, significantly suppressing tumor growth and increasing survival rates. In addition, research on pH-responsive nanoparticles has showcased significant progress in cancer treatment. Swetha et al. [48] and Francesco et al. [49] demonstrated the effectiveness of pH-responsive nanovesicles in drug delivery, especially when loaded with doxorubicin, for potent in vivo and in vitro anti-cancer activity. Marianecci et al. [50] highlighted the importance of surfactants in modulating the pH sensitivity of niosomes, a type of colloidal nanocarrier. Furthermore, the unique ROS-responsive nanomedicine platform has been developed by Li et al. [51] to leverage the elevated ROS levels characteristic of tumors, providing a dual action of oxidative stress-induced apoptosis and chemotherapy for synergistic cancer treatment. Lastly, the exploitation of enzymes in the TME has been successfully demonstrated in the design of enzymatically transformable polymer-based nanotherapeutics. The overactivation of matrix metalloproteinases (MMPs) in tumor-associated tissues catalyzes the transformation of these nanotherapeutics, improving their tissue targeting, retention, and bioavailability while synchronously delivering anti-cancer agents to inhibit malignant progression [52]. Taken together, these studies further support the promising potential of stimuli-responsive nanomedicine in cancer therapy.

The rising prevalence of cancer worldwide necessitates the development of effective therapeutic strategies. In recent years, the therapeutic potential of immune cell-derived nanomedicine, particularly those from macrophages, has been underscored. These nanovesicles exhibit intrinsic long-circulation properties and a natural affinity for cancer tissues, owing to their origin of immune cells that inherently recognize and attack tumor cells [53,54]. Various strategies have been employed to leverage the therapeutic potential of these nanovesicles. For instance, macrophage-derived extracellular vesicles (EVs) have been observed to play a crucial role in the TME, mainly through their ability to shuttle cell-to-cell and organ-to-cell communications by delivering nucleic acids and proteins [53]. However, EVs also present limitations such as low yield, insufficient targeting, and excessively ineffective components, which can be mitigated through engineering [54]. In a novel approach for the treatment of glioblastoma multiforme (GBM), M1-like macrophage-derived extracellular vesicles (M1EVs) were developed to overcome multiple challenges, and were further functionalized with two hydrophobic agents, achieving synergistic immunomodulation, chemiexcited photodynamic therapy (CDT), and hypoxia-activated chemotherapy [55]. This demonstrates the potential of immune cell-derived nanovesicles for targeted, multimodal therapy. Furthermore, combining the properties of liposomes and EVs into hybrid NPs has been shown to enhance siRNA delivery to target cells, illustrating the potential for EV-based systems to enhance the efficacy of RNA interference therapies [56]. These studies highlight the potential of immune cell-derived nanovesicles, particularly macrophage-derived EVs, in developing effective, personalized anti-cancer nanomedicines. Future studies should focus on refining engineering strategies to improve the specificity and efficacy of these nanovesicles in cancer therapy.

Active targeting using folate-targeted nanosystems represents a successful example of active targeting strategies. Folate, a vitamin, is overexpressed on the surface of various tumor cells, making it an ideal ligand for modifying nanocarriers to enhance their tumor cell targeting capabilities [57,58]. Several studies have demonstrated the use of folate-targeted nanosystems in anti-cancer applications. For instance, folate-conjugated polymeric micelles loaded with curcumin exhibited enhanced aqueous stability, sustained drug release, and an efficient intracellular production of ROS, particularly in folate receptor-overexpressing HeLa cells, thereby signifying the critical role of folate conjugation in cell targeting [57]. Folate-targeted liposomes have also been extensively investigated as a delivery system. These liposomes utilize folate attached to phospholipids, cholesterol, or peptides to achieve specific targeting [59], demonstrating the versatility of the folate-targeting strategy. Moreover, Liu et al. [60] reported an enhanced uptake and anti-cancer activity of a folate-targeted bortezomib conjugate in folate receptor-overexpressing cancer cells. These studies collectively highlight the successful utilization of folate-targeted nanosystems in anti-cancer applications, emphasizing their potential for precise targeting and enhanced therapeutic outcomes. Further research in this field could focus on optimizing the design and fabrication of folate-targeted nanosystems and exploring their efficacy in diverse cancer types and models.

Using nanocarriers for the co-delivery of diverse payloads offers a promising strategy to enhance the efficacy and mitigate the toxicity of bioactive compounds. This approach offers the opportunity to achieve synergistic effects by delivering drugs that exhibit co-operative or mutually enhancing effects when co-loaded into nanocarriers [61]. Several successful examples highlight the potential of co-delivery strategies using nanocarriers. For instance, nanoliposomes co-loaded with gemcitabine hydrochloride and paclitaxel exhibited enhanced anti-cancer activity compared to the free drug combination, resulting in decreased cell viability and significant tumor growth inhibition in a mouse model of metastatic breast cancer [61]. Similarly, a nanocarrier system co-delivering Xkr8 short interfering RNA (siRNA) and the FuOXP prodrug demonstrated a significant inhibition of tumor growth in colon and pancreatic cancer models, along with an increased anti-tumor immune response [62]. Moreover, solid lipid NPs co-loaded with microRNA-150 (mR150) and the anti-angiogenic drug quercetin showed strong targeting ability and a prolonged inhibition of choroidal neovascularization (CNV) in a mouse model of age-related macular degeneration (AMD) [63]. Another study utilized trastuzumab-conjugated NPs to co-deliver docetaxel and siRNA against HER2 (siHER2), achieving enhanced efficacy in treating HER2+ breast cancer cells and inhibiting tumor growth in a drug-resistant orthotopic mouse model [64]. Furthermore, a polymeric NP formulation co-loading cisplatin and etoposide demonstrated superior efficacy compared to small molecule chemotherapy in a mouse lung cancer model, indicating the potential of nanotherapeutics in improving chemoradiotherapy outcomes [65]. These studies demonstrate the promise of co-delivery strategies using nanocarriers to enhance therapeutic products by achieving synergistic effects and reducing toxicity. Future research should focus on optimizing co-delivery systems, exploring additional combination therapies, and advancing the translation of nanotherapeutics to clinical applications.

Having explored the above strategies, we now focus on the critical process of drug release, an aspect that determines the effectiveness of any therapeutic nanomedicine. The successful release or production of toxic drugs is a significant factor that determines the overall effectiveness of the therapy. Despite the promising in vitro data often showcasing exemplary release profiles, this process’ in vivo characterization and evaluation has been subpar, as the two environments present drastically different conditions. The recent literature echoes this sentiment, recognizing inadequate drug release as a substantial hurdle in actualizing the therapeutic potential of these delivery systems. Addressing this issue, current innovative approaches have sought to enhance the predictability and control of drug release in vivo. A representative example comes from the study on self-sufficing H_2_O_2_-responsive nanocarriers for anti-cancer drug delivery, which extends beyond in vitro examination [51]. This research showcased a substantial increase in the release of the anti-cancer drug camptothecin (CPT) from hydrogen peroxide micelles (HPMs) compared to that of plain micelles (PMs) within tumor tissues—approximately a 16-fold increase as determined from excised tumors. This approach potentially overcomes the challenge of inadequate drug release that often characterizes traditional drug delivery systems in vivo. Similarly, Li et al. [66] developed a versatile nanoreactor that can initiate the cleavage of oxalate linkages by generating H_2_O_2_, leading to the rapid release of the toxic CPT. Notably, this release mechanism is reliant on an elevated H_2_O_2_ level. Therefore, the problem of inadequate drug release is mitigated as the drug is released in a highly specific and efficient manner, primarily within the tumor site, while the systemic toxicity of chemotherapeutic treatments is reduced. These studies indicate that understanding and exploiting the unique physiological characteristics of tumors, such as elevated ROS levels and acidic environments, can provide novel approaches to enhance in vivo drug release. Future work should continue refining these ROS-responsive delivery systems to optimize drug release profiles and therapeutic outcomes further.

### 2.2. Benefits of NP-Mediated Drug Delivery

Building upon the aforementioned benefits, the subsequent discussion transitions toward the empirical applications of these theoretical constructs. This section elucidates real-world scenarios where incorporating nanotechnology into therapeutic protocols has engendered encouraging outcomes. Such instances substantiate the revolutionary implications of nanomedicine within the broader therapeutic landscape.

Initially, an avant-garde NP platform has been developed to address critical clinical challenges by co-delivering docetaxel and siRNA against HER2 (siHER2) to HER2+ breast cancer cells [64]. This platform has been strategically crafted to guarantee the concurrent delivery of the taxane and siRNA to their target cells, thereby maximizing therapeutic efficacy. Research findings indicate that this NP surpassed the effectiveness of conventional combination treatments in a HER2+ breast tumor mouse model resistant to traditional drugs. Notably, it also proved to be productive in treating brain metastases with a microbubble-assisted focused ultrasound technique. Despite its current experimental status, this platform exemplifies the transformative power of nanomedicine in clinical problem solving and signifies a novel trajectory for cancer therapeutics. Furthermore, numerous NP-based therapeutics for cancer treatment have obtained clinical approval in recent years. A notable example is a nanomedicine platform that encapsulates cisplatin for targeted cancer therapy [67]. SPI-077, liposomal cisplatin, was one of the initial attempts to use nanotechnology to improve cisplatin delivery and reduce its side effects. However, this formulation did not succeed as anticipated due to insufficient release from the liposome. The low on-target activity of SPI-077 led to the failure of this nanomedicine in a Phase II study [68]. This emphasizes the challenge of balancing improved safety and maintained on-target efficacy in developing nanomedicines. NC-6004 is a micelle-based nanomedicine that encapsulates cisplatin in its core [69]. NC-6004 demonstrated a favorable safety profile, and it is noteworthy that this nanomedicine has successfully passed Phase I and II clinical trials [70,71,72]. Encouraging results from a Phase III clinical trial in Japan confirmed its efficacy. NC-6004 is also being investigated with immune checkpoint blockade therapy in a Phase IIb trial for head and neck cancers, indicating its potential in combination therapies [73]. LiPlaCis is another nanomedicine that releases cisplatin following destabilization when anionic lipid membranes are cleaved by sPLA2 [74]. This specific action of sPLA2, which is overexpressed in numerous types of cancer, allows for the targeted release of cisplatin. However, due to stability issues, LiPlaCis’s poor safety profile led to its early termination after Phase I trials. Nevertheless, this formulation has been revised and re-entered into clinical trials, highlighting the perseverance in developing effective and safe nanomedicines for cisplatin delivery [74]. Genexol PM^®^, for instance, is a novel paclitaxel nano-formulation that utilizes passive targeting to enhance its therapeutic potential [75]. The ability of Genexol PM^®^ to exploit the unique pathophysiological traits of tumor vasculature, particularly in metastatic breast cancer, has led to its approval in Korea. Furthermore, its potential in treating other cancers, such as pancreatic cancer, is presently under evaluation in Phase II clinical trials in the U.S. DaunoXome^®^, another example of passive NPs targeting, provides a unique liposomal formulation of daunorubicin, which optimizes the delivery of daunorubicin and has received FDA approval, underscoring the ongoing relevance of passive targeting strategies [76].

These representative nanomedicines, either under clinical trial or approved, have showcased the power of nanotechnology in redefining therapeutic approaches and overcoming the limitations of conventional therapies. The unique benefits of NP-mediated drug delivery, such as enhanced specificity, improved efficiency, and multifunctionality, combined with the potential for personalized treatment, have paved the way for precision medicine.

Enhanced specificity: Due to their customizable physical and chemical properties, nanomedicine can be designed to target specific cells or tissues. Notably, the surface of NPs can be functionalized with ligands or antibodies that recognize and bind to specific receptors overexpressed on tumor cells or immune cells. This targeted delivery not only boosts the therapeutic concentration at the tumor site but also minimizes off-target effects, thereby mitigating systemic toxicity.

Improved efficiency: NPs can be designed to circumvent the physiological barriers that often hinder the efficacy of traditional drug delivery methods, including the use of hydrophilic and hydrophobic molecules. For example, they can enhance the delivery of immunomodulatory agents across the typically impermeable tumor vasculature and dense extracellular matrix. Additionally, nanomedicine is available in various dosage forms and can be administered through multiple routes, such as skin, mucous membranes, lungs, and implants, in addition to oral and injectable forms. Using NPs for drug delivery in tumor immunotherapy offers several substantial benefits. Firstly, NPs can carry a variety of therapeutic payloads, including chemotherapeutic agents, immune stimulants, and other adjuvant therapies. Their small size allows them to navigate the body’s vascular network effectively, infiltrate solid tumors through passive or active targeting, and deliver their cargo precisely to the target cells. This improved delivery efficiency often translates to enhanced therapeutic outcomes. Secondly, NPs can protect drug integrity and biological activity during delivery, thereby boosting drug solubility and bioavailability at the target site, such NPs including doxorubicin, paclitaxel, polyphyllin, and curcumin. They can shield the encapsulated drugs from degradation in the systemic circulation, extending their half-life, enhancing their bioavailability, and reducing their toxicity. In particular, this is advantageous for delivering bioactive molecules such as nucleic acids or proteins, which are inherently unstable and susceptible to rapid degradation in the body. Lastly, NPs can be engineered to release their cargo in a controlled manner over time or in response to specific stimuli such as pH, temperature, or enzymatic activity. This capability ensures the release of pharmacologically active drugs at optimal doses and rates, regulating organ and tissue drug distribution to maintain appropriate concentrations at target sites. This can provide a prolonged therapeutic effect, reduce dosing frequency, and enhance patient compliance. For example, NPs coated with the hydrophilic polymer polyethylene glycol can evade immune system clearance.

Multifunctionality: NPs can be engineered to carry multiple therapeutic agents, allowing the simultaneous delivery of immune stimulants, immune suppressants, and adjuvant therapies. This co-delivery can induce synergistic effects that enhance the overall therapeutic outcome. Furthermore, NPs can incorporate imaging agents, enabling real-time drug delivery and therapeutic response monitoring, and thus optimizing therapy.

**Figure 2:**
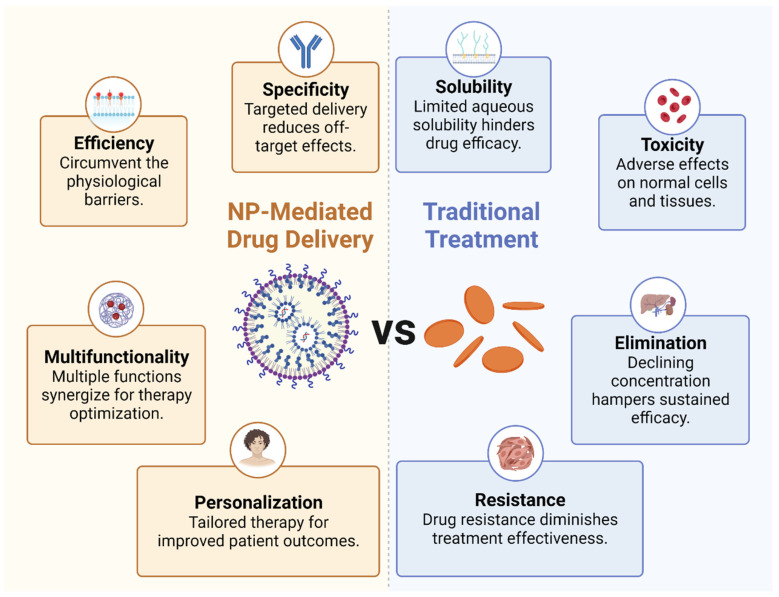
Benefits of NP-mediated drug delivery. The comparison between nanoparticle-mediated drug delivery and traditional treatment methods.

Personalized therapy: Nanomedicines open a promising avenue for personalized therapy. NPs can be customized to a patient’s tumor by incorporating biomarkers or utilizing patient-derived cells. This personalized approach holds the potential to significantly improve therapeutic efficacy and patient outcomes (Figure 2).

## 3. The Promising Role of Nanomedicine in Tumor Therapies

**Figure 3:**
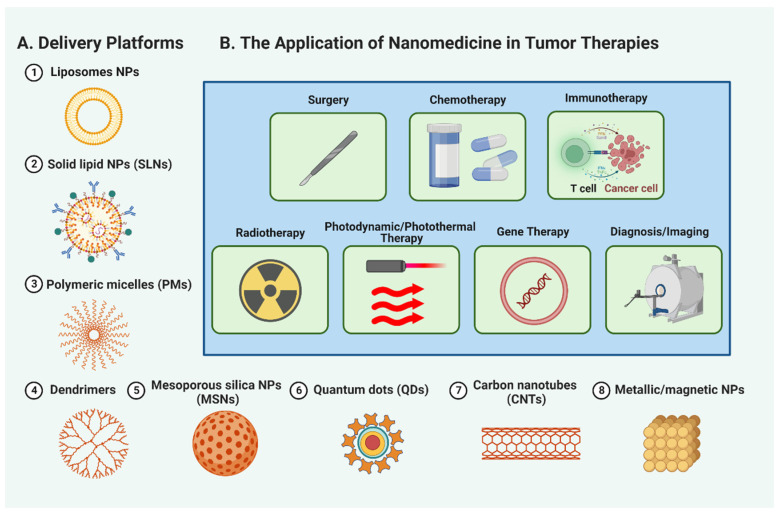
Nanomedicine applications in cancer treatment. A figure (**A**) showcasing the diverse applications of nanomaterials, such as liposomes, solid lipid nanoparticles (SLNs), polymeric micelles (PMs), dendrimers, mesoporous silica nanoparticles (MSNs), quantum dots (QDs), carbon nanotubes (CNTs), and metallic/magnetic NPs, in revolutionizing tumor therapeutics. (**B**) The specific roles of nanomedicine in surgery, chemotherapy, immunotherapy, radiotherapy, photodynamic and photothermal therapy, gene therapy, and tumor diagnosis and imaging, highlighting their significant contributions to reshaping cancer care.

The remarkable properties and capabilities of nanomaterials have catapulted them to the forefront of research in tumor therapeutics. A variety of nanomaterials, including liposomes, solid lipid nanoparticles (SLNs), polymeric micelles (PMs), dendrimers, mesoporous silica nanoparticles (MSNs), quantum dots (QDs), carbon nanotubes (CNTs), and metallic/magnetic NPs, are proving their potential in transforming cancer treatment (Figure 3A, and Table S1). These promising nanomaterials provide targeted drug delivery, improved stability, controlled release, and better penetration into tumor tissues. Additionally, they facilitate multimodal imaging, photothermal therapy, and personalized medicine approaches. Therefore, nanomedicine is an exciting prospect in propelling tumor therapeutics’ evolution and addressing the difficulties associated with conventional cancer treatments. The succeeding sections will discuss the specific roles of nanomaterials in different areas of cancer treatment, including surgery, chemotherapy, immunotherapy, radiotherapy, and more, underscoring their significant contributions to enhancing patient outcomes and reshaping cancer care (Figure 3B).

### 3.1. Surgery

Unlike conventional surgery, which often includes removing diseased tissues and causing potential damage to surrounding healthy ones, nanomaterials offer a valuable tool for efficient and rapid hemostasis, enhancing safety during surgical procedures. For instance, Ma et al. [77] successfully created a nanocomposite porous gel from carbon nanotubes and chitosan derivatives, demonstrating exceptional hemostatic performance on deep traumatic bleeding. Additionally, microscopic tumors, often difficult to discern by the naked eye, significantly challenge surgical effectiveness. However, with the aid of nanotechnology, tumor margins and adjacent critical structures can be illuminated, sentinel lymph nodes can be mapped, and residual tumor cells and micrometastases can be effectively labeled. This considerably improves detection resolution and provides practical guidance for tumor removal [78]. For example, Zhang et al. [79] reported the in vivo assembly of lanthanide-doped NaGdF4-based NIR-II-emitting NPs, notably improving image-guided surgery (IGS) for metastatic ovarian cancer. Furthermore, Moritz et al. [80] engineered a dual-function NP, CLIO-Cy5.5, which serves both as a MRI contrast agent and a near-infrared fluorescent optical probe, demonstrating its effectiveness at delineating tumor margins in orthotopic tumors in hosts. In addition to these benefits, NPs have also been employed to improve the efficiency of cryosurgery, where they can enhance freezing temperature distribution, control the freezing range, and guide ice-ball formation, reducing potential harm to surrounding healthy tissues [81]. Recent research illustrated that introducing MgO NPs into tumor cells notably enhanced their thermal conductivity, thereby improving the freezing effect [82].

### 3.2. Chemotherapy

While traditional chemotherapy is widely utilized, it is not tumor-specific and can damage normal tissue, leading to patient discomfort. It also necessitates repeated administration due to rapid clearance from the body and can result in drug resistance, which reduces patient survival rates [83]. However, NPs can be engineered for specific binding to cancer cells and accumulation at the tumor site, resulting in a highly efficient, low-toxicity therapeutic approach that can overcome tumor drug resistance. Through the exploitation of TME characteristics such as pH, redox gradients, enzyme activity, hypoxia, or specific cell surface receptors, NPs can achieve targeted drug delivery and localized drug release [84]. For instance, lactic acid-modified mesoporous silica NPs have demonstrated specific binding to desialylated glycoprotein receptors on hepatocellular carcinoma cells, thus addressing the drawbacks of non-specific chemotherapeutic drugs. Furthermore, nanocarriers have the potential to deliver multiple therapeutics simultaneously, irrespective of their properties, to maximize treatment efficacy. For instance, Dai et al. [83] reported a self-assembling nanocapsule structure for combined drug delivery, while Huang et al. [85] constructed a system for the co-delivery of paclitaxel and vincristine to brain tumors. These integrated strategies, including vincristine’s multifaceted mechanisms (e.g., apoptosis induction, anti-angiogenesis, and TME modulation), often enhance therapeutic outcomes.

### 3.3. Immunotherapy

Despite the potential of cancer immunotherapies, the response rate remains low, with roughly 10% of patients exhibiting significant improvements. Integrating nanotechnology with immunotherapy could present novel strategies to surmount this barrier. NPs have demonstrated their ability to enhance tumor immunotherapy by augmenting the efficacy of tumor vaccines and modulating the TME to stimulate anti-tumor immune responses. For instance, nanoparticle-based vaccines (nano vaccines) enable the co-delivery of antigens and adjuvants, thus addressing the limitations of conventional cancer vaccines. Notably, nanoparticle delivery to antigen-presenting cells (APCs) has enhanced antigen uptake and stimulated APC activation. Furthermore, emerging nanoparticle-based therapies, including those that utilize metals such as iron, zinc, and copper, have shown potential in tumor immunotherapy. For example, iron NPs can trigger apoptosis in tumor cells through Fenton reactions, promoting a robust adaptive immune response. Zinc NPs have been found to induce immunogenic cell death, leading to systemic immune response stimulation. Copper NPs have been utilized as adjuvants in cancer vaccines, improving the maturation of dendritic cells and antigen presentation. Moreover, nanomedicine can be a platform for combination therapies, co-delivering PD-1/PD-L1 inhibitors with immune stimulants or chemotherapeutic drugs, offering a synergistic approach to address tumor immune evasion. Therefore, integrating nanomedicine and immunotherapy could offer innovative and effective strategies to amplify the body’s immune response against tumors.

### 3.4. Radiotherapy

Radiotherapy, a standard clinical cancer treatment, uses high-energy X-rays to destroy rapidly dividing cancer cells and inhibit tumor growth [86]. Nevertheless, the hypoxic nature of solid tumors due to insufficient oxygen supply makes them 2–3 times more radioresistant than normal tissues are, compromising the effectiveness of the treatment. Furthermore, tumor cells distant from the radiation site receive weak radiation [87]. Nanotechnology has been exploited to create oxygen delivery carriers to combat radioresistance. For instance, fluorocarbon, a temperature-sensitive material with high oxygen adsorption and delivery affinity, has been investigated in this context [88]. Liu et al. prepared tantalum oxide NPs with adsorbed perfluorocarbon, where the NPs focused the radiation energy on the tumor, and the adsorbed perfluorocarbon continuously released oxygen, thereby enhancing tumor oxidation. NPs also show promise as radiosensitizers. For example, Swanner et al. [89] demonstrated the significant cytotoxic and radiosensitizing effects of silver NPs (AgNPs) on triple-negative breast cancer cells in vitro and in vivo. AgNPs have been shown to induce increased DNA damage [90], augment Bax/caspase-3 expression leading to apoptosis, reduce Bcl-2 expression, and decrease catalase, superoxide dismutase, and total GSH levels, thereby enhancing the radiosensitivity of human hepatocellular carcinoma HepG2 cells.

### 3.5. Photodynamic and Photothermal Therapy

Photodynamic therapy (PDT) and photothermal therapy (PTT) are innovative light-based strategies in cancer therapeutics. PDT employs light-activated photosensitizers (PSs) to produce toxic reactive oxygen species (ROS) within tumor cells, causing their destruction [91]. Nevertheless, PDT faces challenges due to tumor hypoxia, the limited tissue penetration of short-wavelength light, the aggregation-induced quenching of PSs, and the potential phototoxicity resulting from systemic PS distribution [92,93,94,95]. These issues can be addressed by nanophotosensitizers, which provide enhanced photophysical properties and facilitate controlled PS delivery [96]. Liang et al. developed a TME-responsive AuNC@MnO2 (AM) nanoplatform for oxygen-enhanced PDT, which can effectively suppress tumor growth and metastasis in metastatic breast cancer. Similarly, a TME-responsive nanoplatform has demonstrated potential to address PDT tumor hypoxia [97]. On the other hand, PTT functions by capitalizing on the high-heat sensitivity of tumor cells and converting light energy into heat, thereby causing intratumoral damage [98]. This approach uses a variety of near-infrared (NIR) nanomaterials, including gold nanorods and carbon nanotubes, among others [99]. However, a limitation of PTT is the induced thermal tolerance in tumor cells due to heat shock proteins (HSPs), necessitating the development of more efficient NIR-absorbing materials [100]. Exciting advancements in both fields have been made. For instance, a porphyrin-based micelle has demonstrated potential in dual-drug delivery and enhanced chemosensitivity under NIR irradiation [101], while gold nanorods have been shown to induce apoptosis in treated cancer cells [102].

### 3.6. Gene Therapy

Gene therapy targets the modification or correction of aberrant genes, incorporating strategies such as gene overexpression or gene silencing via RNA interference (RNAi) [103]. Diverse viral and non-viral vectors have been engineered for the cellular delivery of genetic material. However, many viral gene carriers are restricted due to their severe toxicity and immunogenicity [104]. Safer alternatives include nanocarriers such as gold nanoparticles (GNs), polymer NPs, and lipid NPs for gene delivery. These NPs efficiently transport nucleic acid molecules, shield them from degradation, and enhance drug distribution. For example, the systemic delivery of PTEN mRNA using polymer–lipid hybrid NPs decelerated tumor growth in prostate cancer models [105]. Likewise, RNAi can silence overexpressed oncogenes. Wu et al. [106] suggested PEI-PEG as a promising non-viral carrier for gastric cancer treatment due to its high gene transfection efficiency and low cytotoxicity. Ju et al. [107] took this further by designing a dual receptor-mediated siRNA delivery system for effective, low-toxicity siRNA delivery, achieving specific gene silencing and successfully halting tumor growth.

### 3.7. Diagnosis and Imaging

The importance of early tumor diagnosis in improving survival rates and prognosis is well-established. Traditional non-invasive imaging techniques such as CT, PET, OI, SPECT, and MRI are crucial but pose challenges in terms of sensitivity and specificity, complicating comprehensive disease evaluation [11,108]. Nanotechnology provides innovative avenues in biomedical imaging, introducing multifunctional, tumor-specific devices via NPs such as QDs, liposomes, and AuNPs. These NPs enhance multimodal imaging by integrating with optical, magnetic, and acoustic imaging technologies, providing comprehensive diagnostic information. QDs are particularly noteworthy as exceptional optical imaging nanoprobes, given their unique optical properties and ability to improve cancer detection specificity. The visualization of liposome accumulation within tumor tissues guides the selection of effective targeted therapies. AuNPs, due to their small size and biocompatibility, serve as excellent contrast agents, enabling the detection of early-stage tumors and monitoring tumor cell growth under X-ray irradiation. Furthermore, NPs encapsulated with CT contrast agent iodine yield superior imaging outcomes while minimizing iodine cytotoxicity. With promising results in melanoma and breast cancer models, nanoparticle-enhanced CT imaging and SPECT/CT exhibit tremendous potential for early cancer diagnosis [109,110].

## 4. Prospective View

### 4.1. Prospects and Challenges of Nanomedicine in Tumor Therapeutics

While nanotechnology exhibits substantial potential in tumor treatment by providing unique molecular properties for targeted and less toxic therapies, several hurdles must be surmounted to exploit nanotechnology’s benefits in cancer therapy fully. A significant obstacle includes biological barriers that hinder the accumulation and penetration of nanomedicine into tumors, from blood, and tissue, to the cellular level [111]. At the blood level, the primary hurdles to drug delivery are closely associated with endothelial cells lining the blood vessels. Conditions such as inflammation or cancer often result in poor blood flow, potentially limiting drug delivery. Additionally, the permeability of tumor vasculature and its reliance on the vascular endothelial growth factor (VEGF) can pose considerable challenges to drug delivery [112]. On the tissue scale, barriers may include insufficient vascularization, resulting in hypoxic (low-oxygen) areas that could hinder drug effectiveness and delivery. Elevated interstitial fluid pressures within the TME could impede drugs from effectively penetrating tissues. Additionally, the heterogeneity in tissue characteristics across and within tumors further complicates drug delivery. This heterogeneity might manifest as variations in cell types, genetic mutations, or vasculature, all of which can alter drug delivery and efficacy [111,113]. At the cellular level, the primary challenge stems from the passive nature of most drug delivery methods, which rely on diffusion and concentration gradients for drug transportation from high- (blood) to low-concentration (tumor interstitium) areas, limiting the amount of drug reaching tumor cells [111]. An alternative is active transport mechanisms, utilizing cellular energy stored in the form of ATP or GTP, enabling selective molecular transport across a barrier in the opposite direction to that of the concentration gradient. These barriers, though significant, are not insurmountable. Several strategies, such as vascular promotion therapy, vessel normalization, and iRGD-based drug delivery, are under development to tackle these issues. Nonetheless, transitioning these promising strategies toward clinical reality for cancer patients and other diseases necessitates further research.

In addition, concerns about the toxicity and safety of nanomedicine, including their effects on organs and reproductive systems, are crucial for long-term clinical applications. Moreover, nanocarriers’ drug loading and release control need refinement for optimal therapeutic effectiveness. Barriers to adoption, such as complex synthesis, high costs, limited biocompatibility, and the restricted stability of nanomaterials, impede their broader application in clinical settings. Additionally, the dearth of suitable animal models that closely mirror human tumor diseases poses challenges in translating preclinical findings into clinical trials. Overcoming these obstacles through innovative strategies and extensive research is vital to unlock the full potential of nanotechnology in revolutionizing tumor therapeutics and providing personalized and effective cancer treatments.

### 4.2. Directions for Novel Nanomedicine Development in Tumor Treatment

Nanomedicine holds substantial promise in early cancer diagnosis and treatment, yet it remains in the early stages of development and faces certain limitations that need resolution before obtaining marketing approval. Several key directions should be pursued to advance nanomedicine in tumor therapy. Firstly, lowering the toxicity of nanomaterials and establishing safety evaluation standards are urgent priorities. Secondly, precise nanodrug delivery strategies should be devised to improve delivery efficiency by addressing specific tumor types and overcoming biological barriers. Thirdly, it is crucial to ensure the stability and controlled release of carriers to avoid systemic toxicity and enhance drug delivery efficacy. Simplifying nano-design is also crucial to facilitate clinical translation by resolving complexity challenges. Novel manufacturing equipment and technologies, such as microfluidic technology and non-infiltrating template microprinting, can substantially contribute to the large-scale production of nanomedicine. Lastly, establishing in vivo and ex vivo evaluation models, including human-derived tumor xenograft models and tumor-like organs or microarrays, is vital for a comprehensive evaluation of and to achieve a better correlation between experimental outcomes. Exploring innovative nanomaterial-based drug delivery systems and utilizing advanced techniques such as 3D-printed living tumor models can further boost treatment efficacy prediction, target discovery, and drug development in the future.

## 5. Conclusions

To conclude, nanomedicine, with its potential to address the limitations of traditional therapies, can revolutionize cancer treatment. It leverages nanoparticle-mediated drug delivery for better tumor targeting and drug efficacy, finding applications in various therapies. While challenges such as enhancing biocompatibility, managing drug release, simplifying nano-design, and establishing suitable evaluation models exist, the future of nanomedicine in cancer therapeutics is promising. Continued innovation will help realize its full potential, paving the way for personalized and effective treatments to improve patient outcomes and significantly impact the fight against cancer.

## Data Availability

The study is a review of previously published research, and all data supporting this review are contained within the cited literature.

## Supplementary Materials

The following supporting information can be downloaded at https://www.mdpi.com/article/10.3390/molecules28135145/s1. Table S1: Basic characteristics and Promising roles of common NPs.
