# Supplementary material for: Cancer Nanomedicine: Emerging Strategies and Therapeutic Potentials
Source: Molecules. 2023 Jun 30;28(13):5145. doi: 10.3390/molecules28135145 (PMC10343932; doi:10.3390/molecules28135145)
Supplement: Supplementary file 1 [file molecules-28-05145-s001.zip › molecules-2444040-supplementary.pdf]

Table S1 Basic characteristics and Promising roles of common NPs.

Category	Size (nm)	Particle Structure	Therapeutic Use
Liposomes	50-450	Spherical vesicle with lipid bilayer membrane structure and aqueous core	–Delivery of therapeutics<br>–Gene therapy<br>–Biomedical implants
SLNs	50-1000	Lipid matrix solid at physiological temperature, surfactants and, in some occasions, by cosurfactants	–Controlled release of the incorporated drug<br>–Providing chemical protection for therapeutics<br>–Gene therapy
PMs	10-100	Core-shell structures of spontaneously self-assembled Amphiphilic co-polymers	–Delivery of therapeutics, including pH sensitivity drug release<br>–Imaging contrast agent<br>–Solid tumor therapeutics<br>–Gene therapy
Dendrimers	<15	Highly branched, monodispersed macromolecule with tendrils extending from a central core	–Delivery of therapeutics<br>–Conjugation of chemotherapeutics<br>–Gene therapy
MSNs	50-300	A honeycomb-like porous structure with hundreds of empty mesoporous	–Precisely regulate targeted drug release<br>–Optical, and photothermal properties<br>–Gene delivery<br>–Overcome tumor chemoresistance
QDs	2-10	Heavy metal cores surrounded by a bandgap semiconductor shell	–Optical and fluorescent properties<br>–Biomedical implants<br>–Imaging contrast agent<br>–Therapeutics delivery<br>–Quantum optical semiconductor device
CNTs	10-200	Tiny tubular shapes composed of carbon atoms	–Delivery of therapeutics<br>–Conjugation to antibiotics as an antimicrobial agent<br>–Imaging contrast agent<br>–Tumor imaging and therapeutics<br>–Gene delivery<br>–Efficient and rapid hemostasis, improve the safety of surgery

			–Photodynamic therapy and photothermal therapy
Metallic nanoparticles	1-100	Gold, copper, silver	–Optical, photoelectric, fluorescent, and photothermal properties<br>–Imaging contrast agent<br>–In vivo tumor imaging<br>–Gene delivery<br>–Breast cancer diagnostics<br>–Adjuvants for cancer vaccines<br>-Demonstrated significant cytotoxic and radiosensitizing effects
